# A Comprehensive Approach to Nanotechnology Innovations in Biogas Production: Advancing Efficiency and Sustainability

**DOI:** 10.3390/nano15161285

**Published:** 2025-08-21

**Authors:** Carmen Mateescu, Nicoleta-Oana Nicula, Eduard-Marius Lungulescu

**Affiliations:** National Institute for Research and Development in Electrical Engineering ICPE-CA, 313 Splaiul Unirii, 030138 Bucharest, Romania; carmen.mateescu@icpe-ca.ro (C.M.); marius.lungulescu@icpe-ca.ro (E.-M.L.)

**Keywords:** anaerobic digestion, biomass conversion, biogas, catalysts, nanoparticles

## Abstract

The biochemical conversion of biomass waste and organic slurries into clean methane is a valuable strategy for both reducing environmental pollution and advancing alternative energy sources to support energy security. Anaerobic digestion (AD), a mature renewable technology operated in high-performance bioreactors, continues to attract attention for improvements in energy efficiency, profitability, and long-term sustainability at scale. Recent efforts focus on optimizing biochemical reactions throughout all phases of the anaerobic process while mitigating the production of inhibitory compounds that reduce biodegradation efficiency and, consequently, economic viability. A relatively underexplored but promising strategy involves supplementing fermentation substrates with nanoscale additives to boost biomethane yield. Laboratory-scale studies suggest that nanoparticles (NPs) can enhance process stability, improve biogas yield and quality, and positively influence the value of by-products. This paper presents a comprehensive overview of recent advancements in the application of nanoparticles in catalyzing anaerobic digestion, considering both biochemical and economic perspectives. It evaluates the influence of NPs on bioconversion efficiency at various stages of the process, explores specific metabolic pathways, and addresses challenges associated with recalcitrant biomass. Additionally, currently employed and emerging pre-treatment methods are briefly discussed, highlighting how they affect digestibility and methane production. The study also assesses the potential of various nanocatalysts to enhance anaerobic biodegradation and identifies research gaps that limit the transition from laboratory research to industrial-scale applications. Further investigation is necessary to ensure consistent performance and economic feasibility before widespread adoption can be achieved.

## 1. Introduction

In recent decades, the global energy landscape has undergone complex transformations in a dynamic that has been influenced by the type and availability of resources, energy needs, access to technology and financial availability, as well as political and environmental issues amid increasingly frequent protests by environmental activists. Although fossil fuels are still the dominant energy source, accounting for over 80% of global primary energy consumption, the production of energy from renewable sources has started to become increasingly interesting due to renewable’s potential in reducing carbon emissions and environmental pollution [1].

In 2020 the European Commission approved the European Green Deal, announcing concrete plans to step up climate mitigation policies. This document stipulates that greenhouse gas emission reduction goals for 2030 need to be increased from 40% to either 50% or 55% compared with 1990 levels. This goal requires significant public and private investments in energy efficiency, renewable energy, new low-carbon technologies, and grid infrastructure [2]. The war in Ukraine that broke out in February 2022 has had significant effects on European energy policies and strategies, as it disrupted gas supplies and highlighted the continent’s dependence on fossil fuels. In this context, many countries heavily dependent on imported gas have started to accelerate investments in renewable energy projects, in particular in solar, wind, and bioenergy, as part of the REPowerEU plan, introduced in March 2022, to diversify energy supplies and strengthen energy resilience [3].

Despite the multiple advantages that renewable energy sources offer over fossil fuels in terms of climate change, renewable energy technologies come with various challenges that researchers and developers are trying to address. The challenges in the wind sector relate to the complexity of mathematical models that have to consider meteorological and site variables, component reliability, and also manufacturing and installation costs, etc. [4,5]. Regarding solar energy, there is the major inconvenience of the unpredictability of energy production due to not only variability in solar radiation, but also high installation costs, the reduction in the energy efficiency of the panels over time, the land areas occupied by them, voltage limit violation, etc. [6,7]. Green hydrogen produced through water electrolysis requires large quantities of fresh water, as these supplies are already depleted worldwide; one option in this challenge is the use of seawater, but this is highly corrosive to the anode metal due to chloride ions. Marine and tidal energy involve high costs, have a negative impact on marine biodiversity, and depend on geographical and climatic conditions [1].

Renewable and low-carbon gases play an important role in achieving a reliable net-zero EU energy system by 2050 in a cost-effective manner [8]. Renewable gases produced from biomass (namely biogas and biomethane) or renewable hydrogen can offer solutions to reduce fossil gas import dependency and create synergies between the electricity sector, gas sector, and end-use sectors. Many of the environmental, economic, and social benefits that the bioenergy sector can bring are unique compared with other renewable energy sources; e.g., it contributes to energy security and soil health and eliminates organic waste through controlled processing; the resulting carbon dioxide is biogenic in nature, captured, and used in multiple industrial applications, etc. [9].

Renewable gases and liquids produced from biomass (biogas, bioethanol, biodiesel, and renewable and low-carbon biohydrogen) can offer green solutions to store the energy produced from variable renewable sources, exploiting synergies between the electricity sector, gas sector, and end-users. Biomass into bioenergy conversion can occur in two different ways: thermo-chemical conversion (combustion for heat or electricity, pyrolysis into bio-oil and coal, catalytic liquefaction into marketable liquids, and gasification into synthesis gas) and biochemical conversion for the production of biogas, biohydrogen, and bioethanol, the latter following three different types of processes: anaerobic digestion, alcoholic fermentation, and photobiological reactions. All biochemical processes involve microorganisms or enzymes to transform biopolymers into gaseous or liquid biofuels [10]. In addition to enzymes that are biological catalysts in biochemical processes, studies have shown that various other mineral catalysts, such as zeolites, if added to the fermentation substrate in fermentative processes, can facilitate microbial metabolism and increase biomass conversion yields, thus accelerating the production of biomethane, biohydrogen, or liquid biofuels [11]. The bioenergy field can be accelerated and made more efficient by improving catalytic conversions.

Anaerobic digestion is a mature method recommended for the valorization of biomass with a high moisture content toward biogas and further to biomethane by upgrading. The biotransformation of wet biomass waste into green methane is not only an excellent practice for reducing environmental pollution and a feasible option for capitalizing on alternative energy sources in achieving energy security goals [12], a nutrient-rich by-product, which can be used as a biofertilizer, is also obtained simultaneously with the fuel gas via anaerobic digestion. Anaerobic digestion is a complex biochemical process, which is carried out by microorganisms in the absence of oxygen and goes through the following four biochemical steps: (a) hydrolysis of substrate macromolecules (polysaccharides, fats, and proteins) into smaller molecules (sugars, long-chain fatty acids, and amino acids) under the action of extracellular enzymes secreted by hydrolytic bacteria; (b) biochemical transformation of the biomolecules resulting from hydrolysis into volatile fatty acids, alcohols, fermentation gases (ammonia, carbon dioxide, and hydrogen), and other by-products by acidogenic bacteria (acidogenesis); (c) conversion of short-chain volatile fatty acids and alcohols and aldehydes into acetic acid, carbon dioxide, and hydrogen by acetogenic bacteria (acetogenesis); and (d) conversion of acetic acid, carbon dioxide, and hydrogen into biogas consisting mainly of methane and carbon dioxide by archaea microorganisms (methanogenesis) [13]. In this complex process, various microbial consortia must coexist in the dynamic fermentation mass, in a biochemical balance that allows the four consecutive stages to be completed, although the microbial strains disturb each other as they have different optimal growth conditions. The dynamics and diversity of microbial consortia makes anaerobic digestion a very unstable biochemical process, with the risk of process failure in the event of any slight variations in environmental parameters.

Extensive research has been undertaken in recent years to develop and implement various pre-treatment techniques for recalcitrant biomass substrates, especially those with high lignin content, to facilitate the biodegradation of complex biopolymers and the access of bacteria to organic compounds necessary for specific metabolisms. Classical pre-treatment techniques (mechanical, physical, chemical, and biological and their combinations such as physico-chemical, bio-physico-chemical, mechano-chemical, and thermo-chemical) have been developed, as well as less conventional ones, such as gamma-irradiation, supercritical CO_2_ explosion, acceleration of microbial metabolism by nutrient supplementation, including the use of various nanoparticles, such as trace metals (e.g., iron, cobalt, nickel, and silver), metal oxides (e.g., Fe_3_O_4_, and carbamide-capped Fe_3_O_4_, ZnO, and CeO_2_), silica nanogels, etc. [14,15,16,17].

Since anaerobic digestion with biogas production, which allows the use of locally available organic matter, is the biochemical process already implemented on an industrial scale globally, there is a growing interest in increasing the energy efficiency and profitability of biogas plants. This study will focus on a less addressed strategy for improving the efficiency of the global anaerobic digestion processes, more specifically the use of nanoparticles as catalysts for biochemical reactions. The need for this review paper stems from the fact that very few studies have been conducted and reported in the literature on the effect of the type and concentration of nanoparticle-type micronutrients in biogas reactors, which would lend confidence to this approach to increasing biogas production and stabilizing overall processes, thus increasing the profitability of biogas facilities. The paper aims to increase the level of knowledge on the use of nanoparticles in catalyzing anaerobic biochemical reactions and to provide confidence in this relatively under-researched field, although, as an individual approach, the waste-to-biogas and nanomaterial fields are being researched in depth and developed at an application level. No less importantly, the challenges of this interdisciplinary approach will be brought to light and recommended to scientists as feasible study directions for the economical and efficient use of nanoparticles in future research.

## 2. Methodology

**Figure 1:**
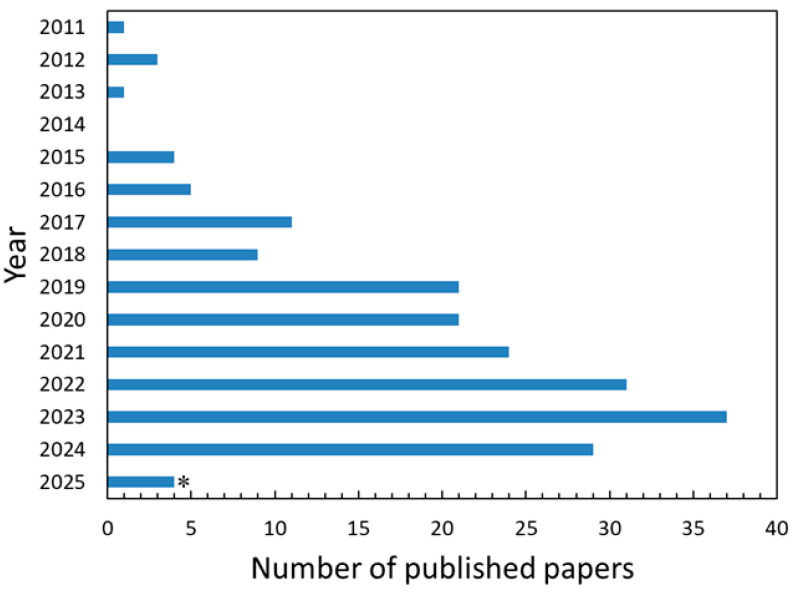
Number of published papers, after manual selection, related to the use of nanoparticles in anaerobic digestion and biogas production; * up to the date of publication.

To gather relevant publications for the review, a Web of Science (WoS) database search was conducted using the terms “Anaerobic digestion” AND “Biogas” AND “Nanoparticle” with the search limited to English language articles, excluding review articles, books, and conference papers; this initial search yielded 321 articles, which was further refined through a manual selection process based on title and abstract relevance to the search terms, resulting in a final set of 201 publications from 2011 to 2025 (Figure 1).

**Figure 2:**
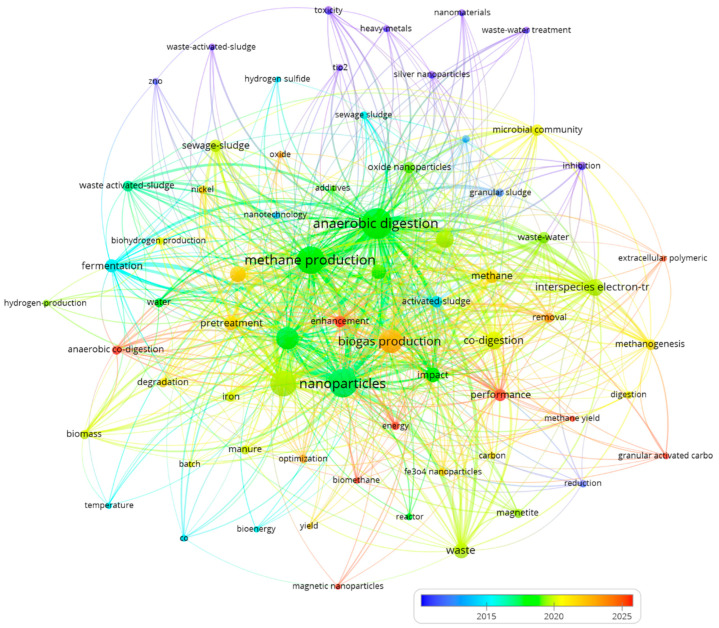
Holistic map related to keyword co-occurrence (Keywords used at least 5 times) using VOS viewer 1.6.20 software version.

Using the same results obtained from WoS database, Figure 2 presents a network visualization illustrating the co-occurrence of keywords in research publications related to “nanoparticles,” “anaerobic digestion,” and “biogas,” revealing that the central nodes of “nanoparticles,” “anaerobic digestion,” “biogas production,” and “methane production” are strongly interconnected. This indicates a significant focus on the relationship between nanoparticles and their enhancement of biogas and methane production within anaerobic digestion processes. The network also displays clusters of related keywords concerning inputs and processes like “sewage sludge,” “pre-treatment,” and “co-digestion,” specific nanoparticles such as “magnetite” and “Fe_3_O_4_,” outcomes and impacts like “methane yield” and “enhancement,” and microbiology-related terms like “microbial community” and “methanogenesis,” collectively suggesting a growing, multidisciplinary field investigating the potential applications of nanoparticles to improve the efficiency and sustainability of biogas production, with ongoing research that could explore long-term impacts and optimize nanoparticle use.

## 3. Biochemical Conversion Pathways to Biogas

The anaerobic bioconversion of organic materials to the final stage of biogas and carbon dioxide is a process that takes place strictly in the absence of oxygen through some very diverse and complex biochemical pathways, where rich consortia of fermenting microorganisms (archaea, bacteria, and fungi) work together under environmental conditions that can ensure optimal microbial growth [18]. These conditions are mainly the acidity of the fermentation mass, the temperature of the substrate, the hydrostatic pressure, and the concentration of nutrients [19].

**Figure 3:**
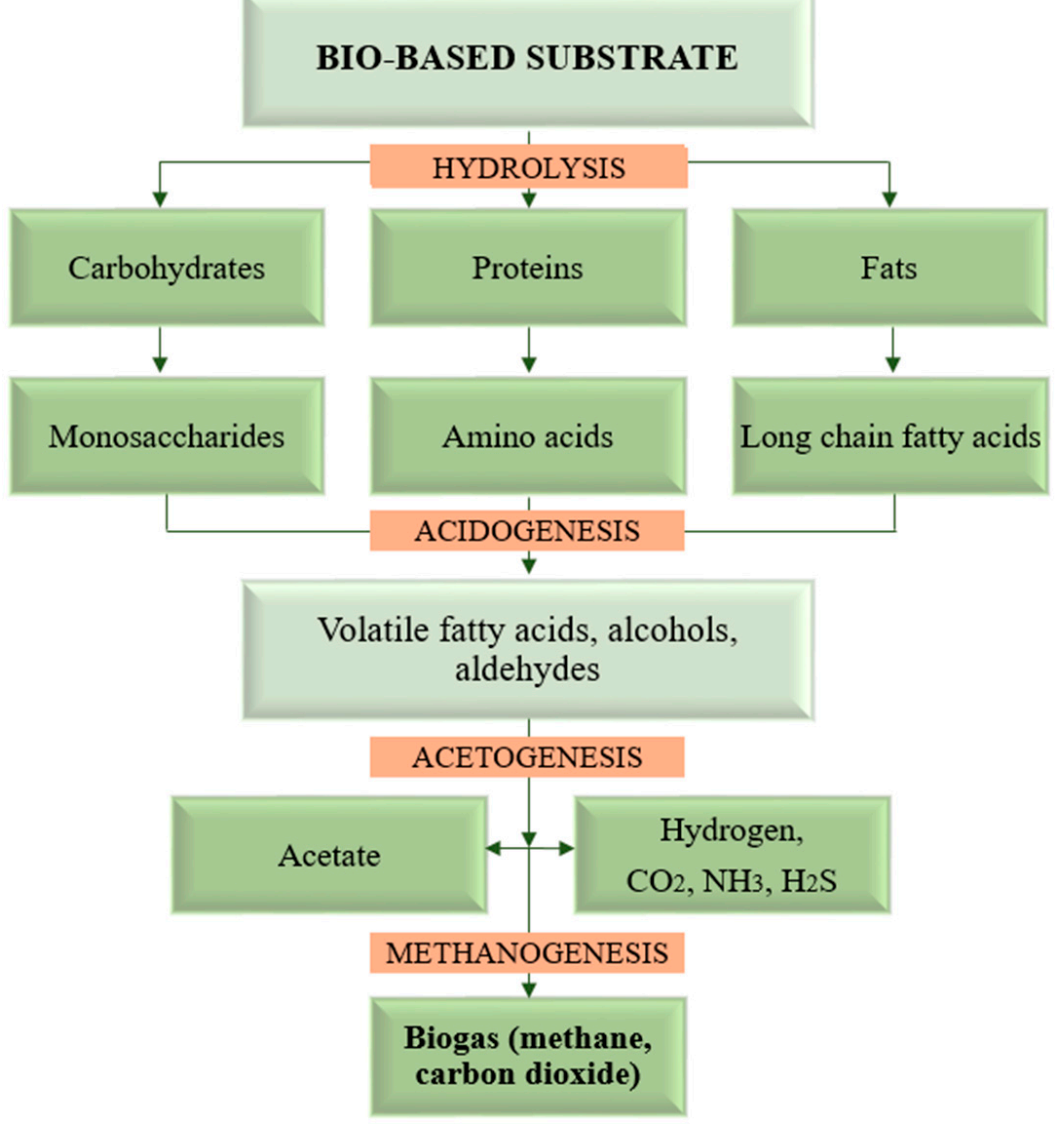
Stages of biomass-to-biogas transformation.

Although there are still some biochemical pathways in this complex process that are still unknown, it is generally agreed that anaerobic digestion to biogas successively goes through the following four main transformation stages: hydrolysis, acidogenesis, acetogenesis, and methanogenesis [20]. The simplified graphical representation of the biomass-to-biogas decay, starting from organic macromolecules to the final decomposition gases, is shown in Figure 3.

*Hydrolysis*, or solubilization, is the first stage of the decomposition process where biopolymers with a large molecular structure (carbohydrates, starch, proteins, and fats) are converted into soluble organic compounds with smaller molecules (monosaccharides, amino acids, peptides, and fatty acids) by means of extracellular enzymes secreted by hydrolytic bacteria. Following this process, the organic compounds with smaller molecules are able to diffuse through the cell membrane of acidogenic microorganisms to be metabolized [10,20]. Hydrolytic microorganisms are very resistant species, adaptable to different environmental conditions as well as to sharp changes in substrate acidity (pH in the range of 4–11), but they have a very low efficiency in the decomposition of recalcitrant biopolymers such as lignin, thus increasing the duration of the global decomposition processes. Therefore, the addition of specific enzymes can considerably improve the speed of this process step [21].

*Acidogenesis* is the biochemical step that is assumed to occur most rapidly, in which intermediate organic products cross cell membranes and are broken down by acidogenic microorganisms, thus resulting in volatile fatty acids, alcohols, and aldehydes. The ratio at which these intermediates are generated depends on the technical and operational parameters of the digester but also on the type of substrate. Mainly, these intermediates are C2-C4 organic acids in the form of salts (acetate, propionate, and butyrate), along with other oxygenated compounds. The fast growth of acidogenic microorganisms, with a minimum doubling time of 30 min. [22], correlated with the high level of organic acids that accumulated in a relatively short time and were often responsible for digester failure, if this was not attributed to certain accidental inhibitors (detergents, antibiotics, etc.).

*Acetogenesis* is the stage that proceeds with slower kinetics than acidogenesis (the doubling time of acetogens is 1.5–4 days). Acetic acid formed in acidogenesis from glycerol and the oxidation of long-chain fatty acids is used in acetoclastic methanogenesis, while higher fatty acids, alcohols, and other intermediates are mainly converted into acetic acid, hydrogen, and carbon dioxide, along with formic acid and methanol. Interestingly, in this stage, hydrogen is formed in large quantities, but paradoxically, the increased partial pressure is detrimental to acetogenic microorganisms. In the microbial consortium, there are also hydrogenotrophic methanogens that rapidly consume hydrogen from the system and prevent the inhibition of acetogens [23].

**Figure 4:**
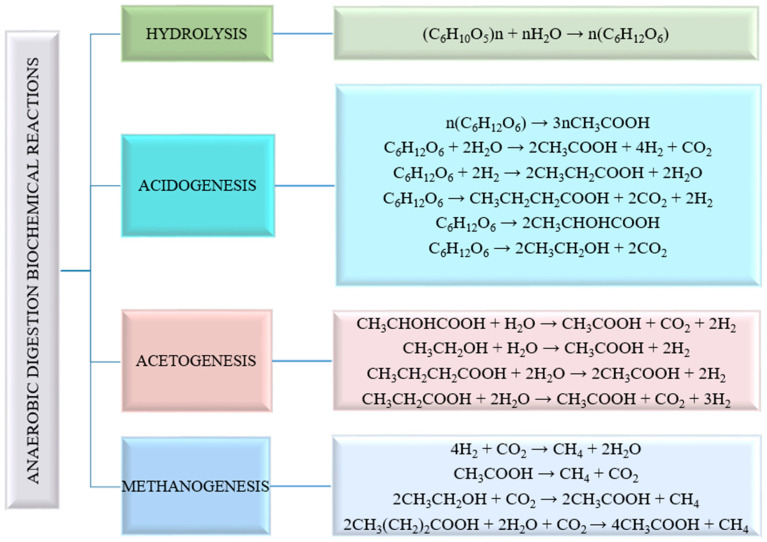
Biochemical conversion pathways to biogas.

*Methanogenesis* is the final stage of the anaerobic digestion process, which is the slowest stage and requires higher pH compared with the previous stages [24]; the regeneration time of methanogenic microorganisms is 5–16 days. In this stage, the final gaseous product, called biogas, is generated, consisting mainly of methane (50–75%) and carbon dioxide (20–40%), along with small amounts of hydrogen sulfide, ammonia, carbon monoxide, nitrogen, and water vapors [25]. Methane-producing archaea are diverse; therefore, the metabolic pathways for methane formation are different. Some species use acetic acid to produce methane (2/3 of methane is produced by acetotrophic methanogens), while others use hydrogen and carbon dioxide (1/3 of methane is produced by hydrogenotrophic methanogens); but a few other species use compounds such as formiate, alcohols, and amines in the methane biosynthesis process. Methanogens are obligate anaerobic species that are extremely sensitive to environmental conditions as well as to small variations in environmental parameters (temperature, pressure, pH, traces of oxygen, inhibitors as excess ammonia, sodium, etc.) [26]. The main biochemical processes and the reaction products of anaerobic digestion to biogas, according to the statements in references [10,27], are depicted in Figure 4.

For a fluent understanding of the complex biochemical pathways, the anaerobic digestion process is presented as occurring in stages; in reality, these distinct stages occur simultaneously, with different consortia of microbes coexisting and working together to transform biomass into biogas. The complex nature, dynamics and different environmental preferences of the microbial consortia involved in anaerobic digestion, with widely varying microbial growth rates, make the overall process of biomass conversion into biogas a very unstable one [28].

## 4. Current Status and Future Prospects to Enhance Waste-to-Biogas Conversion Efficiency

In waste-to-biogas applications, the type and quality of biomass, the different rates of the biochemical steps mentioned above, and also the different sensitivities of the microbial groups involved in the processes are obstacles that can impede the anticipation of biogas production and, implicitly, the profitability of the investment [29]. Fresh or residual plant biomass constitutes an important share in the global renewable energy resource but, in the case of biogas bioreactors lignin in plant tissue, acts as a barrier to the access of hydrolytic microorganisms to digestible chemical compounds, such as cellulose and hemicelluloses [30]. Biopolymer hydrolysis or solubilization is the speed-determining step of the whole process, and the more recalcitrant biomass is present in the substrate, the more time will be required for the substrate decomposition. On the other hand, methanogenesis is the slow stage but most sensitive in the case of processing readily biodegradable substances [31] and can lead to process failure in case of sudden changes in acidity and temperature or the generation of inhibitory substances [25]. The operational parameters responsible for biogas production, including the substrate type, the carbon-to-nitrogen ratio, the use of co-digestion materials, feedstock pre-treatment and the techniques used for this purpose, the technical characteristics of the reactor, the fermentation temperature, substrate acidity and the organic loading rate, stirring, concentrations of volatile fatty acids and free ammonia, and the type of inoculum used, should be carefully monitored and optimized to improve biomass conversion efficiency and reduce the costs associated with this technology [32].

Carbohydrate-rich substrates cause the accumulation of volatile fatty acids to rise beyond the capacity of the bacteria to metabolize acids (mainly acetic, propionic, and butyric acids); hence, the pH of the fermentation mass may drop below 6.6 and inhibit the activity of methanogens. It was reported that the highest biogas production occurred when the volatile fatty acids were low, usually below 2 g/L [33].

Fan et al. [34] used nano-bubble water (NBW), that is, water containing bubbles with diameters ranging from a few nanometers to several hundred nanometers, to mitigate volatile fatty acid inhibition, and they achieved improved methane production by 12.2%. This can become a practical and ecological method also applied in biogas technologies, in addition to applications in agriculture, medicine, and environmental engineering, because the production of NBW is inexpensive, does not involve energy consumption, and does not generate any polluting chemicals.

Anaerobic digestion of organic materials with high nitrogen content, such as animal manure, sewage sludge, and algal biomass, can lead to the accumulation of free ammonia in equilibrium with ammonium ions in the fermentation slurry; ammonium and ammonia are toxic to methanogens and decrease biomethane production. Ma et al. [35] revealed that in fermentation substrates under ammonia inhibition conditions, the stabilization of methanogens and, implicitly, the increase in methane production by 30,53% could be achieved by adding 3 g/L of lignin-based carbon materials synthesized by hydrothermal carbonization to the digester [34].

Biomass solubilization for advanced degradation of organic compounds can be achieved through various pre-treatment techniques, which could be mandatory steps for materials containing recalcitrant biopolymers [36]. Pre-treatment results in a reduction in the crystallinity of coarse cellulose in the aqueous suspension, a reduction in particle size, and an increase in surface area for easier access of fermentative microorganisms to digestible fragments, thus leading to a higher efficiency in sludge biomethanization [37].

Conventional and innovative chemical, physical, and biological pre-treatment techniques have been reported in the literature to change the physical–chemical structure and improve the biodegradability of various biomass. A combination of one or more techniques can be applied to achieve better solubilization of the substrate to be fermented [29]. Table 1 shows some common pre-treatment techniques capable of increasing biomass bioconversion to biogas [10]. Biomass pre-treatment increases the cost of the overall bioconversion process as it leads to increased energy consumption and, in some cases, material consumption, with or without generating waste or polluting wastewater. Thus, the correct selection of the pre-treatment method is essential to achieve increased biogas production with increased sustainability and profitability. Also, the choice of a particular method should take into account the nature of the substrate, particularly its chemical composition [38].

Pre-treatment techniques have largely addressed the fragmentation of molecular structures to accelerate the microbial decomposition of poorly digestible compounds, adjusting the acidity of the fermentation medium, preventing excess ammonia generation, and, to a lesser extent, supplementing the biomass with macronutrients as well as micronutrients for enzyme activity; these nutrients must remain within an optimal range for the metabolisms of the microbial species in the system since even small variations in the concentrations of these nutrients could inhibit methanogenesis and lead to poor biomethane yields [39].

Scientific studies carried out over the past few years have shown that supplementing the substrate with nutrients at a nanoscale would have better effects on biomethane yield compared with supplementing with micronutrients, most likely due to the higher surface area/volume ratio, and also the higher reactivity of nanoparticles on the enzyme’s activity compared with microparticles of the same class [39,40]. The use of nanoparticles in biogas technologies proves to be a promising solution for improving not only the bioconversion to biogas by increasing the process stability and the production and quality of biogas, but also the quality of the by-products resulting from the digestion process.

## 5. Nanotechnology and Biogas Production

**Figure 5:**
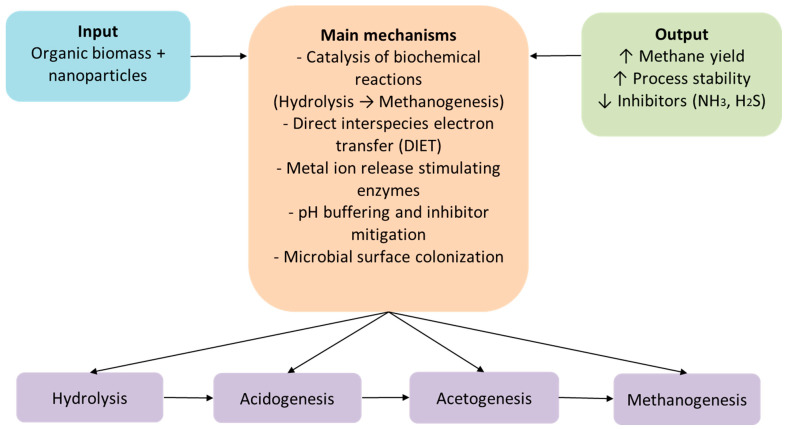
Main mechanisms by which nanotechnology enhances anaerobic digestion.

Nanotechnology offers some synergistic pathways to enhance the performance of anaerobic digestion. When organic biomass is enriched with specific nanoparticles, these substances actively interact with microbial communities and biochemical processes, catalyzing significant steps of anaerobic digestion, from hydrolysis to methanogenesis [41,42]. They also contribute to fast substrate decomposition, leading to net conversion efficiency improvement (Figure 5). One of the most widely accepted and studied mechanisms by which nanoparticles contribute to improving biogas production is the mechanism of direct electron transfer between microbial species (DIET—direct interspecies electron transfer) [43,44,45]. This is based on the ability of conductive nanoparticles, such as magnetite, carbon nanotubes, or graphene, to act as electrical bridges between the microbial communities involved in the anaerobic digestion process. Through these conductive “bridges,” electrons are transferred more quickly and efficiently from fermentative bacteria to methanogens, without the need for the accumulation or conversion of unstable chemical intermediates such as molecular hydrogen or volatile acids. This accelerates the metabolism of microbial consortia, reduces the risk of inhibition, and, implicitly, significantly increases methane yield. In addition, the DIET mechanism provides greater stability to the anaerobic fermentation process, even under stressful conditions or variations in environmental parameters, making it very attractive for industrial-scale application in modern biogas production technologies [46,47].

The integration of nanomaterials into biogas production, particularly through anaerobic digestion, marks a major breakthrough in the renewable energy sector. Nanotechnology has been recognized for its potential to enhance the efficiency and yield of biogas production by modifying the microbial processes involved in anaerobic digestion. The application of nanomaterials, such as metal nanoparticles (MNPs), carbonaceous nanomaterials (e.g., graphene and carbon nanotubes) has been shown to improve the biochemical methane potential (BMP) of various substrates, thereby increasing the overall biogas yield. For instance, studies have demonstrated that magnetic nanomaterials can enhance biogas production from municipal wastewater treatment by improving the degradation of organic matter and facilitating methane generation [41]. Furthermore, the use of trace metal solutions, including iron and copper, has been linked to increased methane production, highlighting the role of nanomaterials in optimizing anaerobic digestion processes [41].

The enhancement of anaerobic digestion through the addition of nanomaterials has been the subject of numerous studies (Table 2), which collectively indicate that these materials can positively influence various parameters of the digestion process. For instance, a mini-review highlighted that adding nanomaterials in batch anaerobic digestion systems often resulted in notable enhancements in biogas production [42]. This is due to the ability of nanomaterials to improve microbial activity and substrate degradation, thereby facilitating a more efficient digestion process. Moreover, the critical review of nanomaterials for biogas augmentation emphasizes the diverse applications of nanoparticles, nanosheets, and other nanostructures in enhancing bioenergy production [48]. The results suggest that the physico-chemical characteristics of nanomaterials enable them to interact effectively with both substrates and microorganisms, leading to improved digestion kinetics and biogas yields.

In addition to enhancing biogas production, nanomaterials can also contribute to the reduction in environmental impacts associated with anaerobic digestion. For instance, the application of photocatalytic pre-treatment methods using nanomaterials improved the degradation of lignocellulosic substrates, resulting in enhanced biogas yield while simultaneously reducing the environmental footprint of waste management practices [49].

**Table 2:** Recent results related to the use of nanoparticles for biogas production.

Nanomaterials	Nanomaterials Properties	Substrate	Main Results	Reference
**Multiwall carbon nanotubes (MWCNTs)**	- Diameter: 10–20 nm - Length: 3–8 µm - Purity: 99%	Food waste	- Increase in biogas production by 33.35% compared with control; - Decrease in total solid content by 25.95% compared with control.	[50]
**Graphene nanoparticles**	- Thickness: 2–4 nm - Lateral size: 5 µm - Purity: 99%	Food waste	- Increase in biogas production by 81.16% compared with control; - Decrease in total solid content by 24.95% compared with control.	[50]
**Nano-C60**	- NPs size: 90 nm - Purity: 99.9%	Chicken manure with sulfamethoxazole (SMX)	- SMX degradation rate (day 5) reached 99.81%; - Cumulative biogas production increased by 35.4%.	[51]
**Fe_3_O_4_NPs and MWCNT**	- NPs’ size: 50–100 nm (Fe_3_O_4_NPs); 2 µm long and external diameter of 10–30 nm (MWCNT) - Concentration: 3.2 g/L (Fe_3_O_4_NPs) and 3.5 g/L (MWCNT)	Chicken manure	- Reactors including either Fe_3_O_4_NPs or MWCNTs yielded a greater amount of CH_4_, higher for combined NP systems; - NPs accelerated the breakdown of acetate and butyrate, and their combined presence further enhanced the utilization of these VFAs in the methane-production process; - NPs had a combined effect on the functioning and well-coordinated action of anaerobic communities.	[52]
**Fe_3_O_4_@biochar**	- Fe_3_O_4_ synthesized by co-precipitation method - NP concentration: 100, 200, and 300 mg/L - NPs’ size: ~18 nm - Saturation magnetization of Fe_3_O_4_@BC: 55.75 emu/g	Vegetable waste	- Enhanced microbial diversity and abundance; - Increased biogas production rate and reduced the accumulation of volatile fatty acids (VFAs); - Optimum concentration: 200 mg/L Fe_3_O_4_@biochar.	[53]
**Fe_3_O_4_ NPs**	- Different sizes NPs: 176 nm, 164 nm, 184 nm - Cubic morphology and uniform distribution	Swine manure after bioconversion by black soldier fly larvae and co-digestion with corn straw	- Reduced the lag phase and increased the activity of methanogenic bacteria; - Gas production was dependent on NPs’ size, with those of 184 nm being most efficient; - Increased the methane content.	[54]
	- NPs size: 90 nm - Purity: 99.8%	Chicken manure with sulfamethoxazole (SMX)	- SMX degradation rate (day 5) reached 99.64%; - Cumulative biogas production increased by 130.7%.	[51]
	- Fe_3_O_4_ NPs were biosynthesized form *Vicia faba* L. peels - NP concentrations: 25, 50, 75, 100 ppm - Irregular shape NPs with an average diameter of 97.2 nm - Saturation magnetization: 25.48 emu/g	Wheat straw underwent pre-treatment with potassium hydroxide + Cattle rumen fluid	- Daily biogas yield: 9.96 mL/g vs. that of the control, 12.20, 14.66, 17.24, and 19.62 mL/g vs. that of the digester’s inclusion with 25, 50, 75, and 100 ppm of MNPs (after about 18 days); - The maximum methane yield was obtained for 100 ppm NPs (169.6 mL).	[48]
**Iron based nanoparticles (nano zero-valent iron and Fe_3_O_4_—magnetite)**	- FeNPs—100 nm, 99.9% purity - Fe_3_O_4_—98% metal basis	Food waste	- Carbon degradation pathway changed by dosing metallic nanomaterials; - Nano zero-valent iron was superior to Fe_3_O_4_ in boosting CH_4_ yield.	[55]
**Polypyrrole (Ppy) Fe_3_O_4_ NPs**	- NP concentration: 0, 20, 40, 60, 80 mg/L;	Sewage sludge and wheat straw	- The maximum biomethane yield of about 50% higher than the control was obtained for 40 mg/L PPy/Fe_3_O_4_ NPs; - Sustainable biomethane production was optimized using 50.24 mg of nanoparticles and 24.04 mg of NaOH, resulting in a 198% increase in biomethane yield while simultaneously reducing environmental and economic impacts by 87–95%.	[56]
**FeNPs**	- Two class of NPs: laboratory synthesized (Type I) and derived from industrial recycled NPs and slag (Type II) - NPs’ size: 15 nm (Type I) and 10 nm 9 (Type II) - NP concentration: 0, 25, 50, 100 mg/L	Raw manure	- Both NP types boosted biogas (up to 123%) and methane (up to 108%) production.	[57]
	- FeNPs were obtained by chemical reduction - NPs’ size: 10–20 nm with a worm-like shape.	Pig slurry	- Methane content of biogas reached values of 88%; - A total of 98.6% of the residual FeNPs was effectively retrieved from the sludge through magnetic separation for reutilization.	[58]
	- NPs were obtained by chemical reduction of FeCl_3_ × 6H_2_O - FeNPs had a core–shell structure, assembled in a chain-like structure	Co-digestion of waste sludge with aloe vera waste		[59]
**Silver nanoparticles (AgNPs)**	- NPs’ size: 15 nm - AgNP concentration: 40 mg/L	Sewage sludge	- AgNP addition increased methane yield by 15% in contrast to the AD of sewage sludge alone (control); - Addition of silver into feedstock did not negatively impact the process stability.	[60]
**SiNPs and Ag@SiNPs**	- Mesoporous materials with a pore size of 7.64 nm (SiNPs) and 5.15 nm (Ag@SiNPs) - Surface area: 727 m^2^/g (SiNPs) and 142 m^2^/g (Ag@SiNPs) - Concentration of NPs: 50 mg/L	Landfill leachate	- Degradation efficiency for Vs: 58.4% (SINPs) and 65.08% (Ag@SiNPs), compared with 53.9% for the control; - Chemical Oxygen Demand (COD) degradation: 37.2%, 48%, and 49.1% for the control, SiNPs, and Ag@SiNPs; - Acetoclastic methanogens was the route for transformation of organic compounds to biomethane; - Ag@SiNPs stimulated the activity of microbial species involved in the methanogenesis process.	[61]
**Cobalt (CoNPs) and Ni (NiNPs) nanoparticles**	- Nanoparticles were synthesized by the reduction method - CoNPs and NiNPs had a homogeneously spherical shape - NPs’ size: 17 (NiNPs); 28 (CoNPs) nm	Raw manure	- The nanoparticles increased both biogas and methane yield, reduced lag phase, and biostimulated and increased the activity of methanogenic bacteria.	[39]
**NiO Nps**	- NPs were synthesized by the co-precipitation method - NPs’ size: 15–45 nm - Multiform NPs’ shape: spherical, oval, hexagonal, tubular - Surface area: 70.1 m^2^/g - Used concentrations: 5, 10, 15 mg/L	*C. linum;* control: *C. linum* + cow manure	- Increased the biogas yield up to 384 mg/L Vs (5 mg/L NPs) and 380 mL/G Vs (15 mg/L NPs) at 1.5 g *C. linum*; - A total of 94.81 mL/g Vs biomethane generation (15 mg/L NPs and 1.5 g *C. linum*); - NiONPs significantly reduced key environmental impacts—compared with the control (*C. linum* and cow manure without NiONPs), due to biomethane production	[62]
**NiO, CoO, and Fe_3_O_4_ nanoparticles**	- NPs’ size: 15–45 nm (NiO), 50 nm (CoO) and 20–30 nm (Fe_3_O_4_) - Used concentrations: 1, 10, 100 mg/L	Primary sludge and waste-activated sludge of municipal wastewater	- Increased biogas yield by 26.94% (1 mg/L NiO), 21.12% (1 mg/L CoO), 23.41% (10 mg/L Fe_3_O_4_), and 14.29% (100 mg/L Fe_3_O_4_) and methane yield by 39.04%, 30.37%, 35.59%, and 22.65%; - NPs reduced the time to reach peak biogas and methane yields compared with the control; - H_2_S content reduced with increasing of NP concentration; - Addition of NPs improved the degradation of organic content.	[63]
**Fe, Ni, Co nanoparticles**	- NPs’ size: 30.0–80.9 nm - NP concentration: 1000 mg/L Fe, 120 mg/L Ni, and 54 mg/L Co	Poultry litter	- NPs increased the biogas production by 23.7% and led to 56.3% less cumulative H_2_S production.	[64]
**Fe-Co-Zn trimetallic nanoparticles (TMNPs)**	- TMNPs were synthesized by the co-precipitation method	Palm oil mill effluent (POME)	- The POME-based AD process increased biogas production by 85% compared with the blank AD process	[65]
**Fe_3_O_4_, CoFe_2_O_4_, and NiFe_2_O_4_ NPs**	- NPs’ size: 5.7 nm (Fe_3_O_4_), 4.9 nm (CoFe_2_O_4_), and 3.5 nm (NiFe_2_O_4_) - NPs: spherical shape, slightly aggregated, and good dispersion - Saturation magnetization: 56.9 (Fe_3_O_4_), 34.5 (CoFe_2_O_4_), and 17.9 (NiFe_2_O_4_) emu/g - Used concentrations: 100 mg/L	Cow dung	- Cumulative biogas yield (HRT;50 days): 4929 mL (Fe_3_O_4_), 5155 mL (CoFe_2_O_4_), 4020 mL (NiFe_2_O_4_), and 2337 mL (Control)	[66]
**Sn-Mn-Fe TMNPs**	- TMNPs were synthesized by the co-precipitation method using electronic waste - NPs’ size: 50–100 nm - NPs’ shape: spherical - Surface area: 70.1 m^2^/g - Used concentrations: 20, 50, 100 mg/L	Fresh cow manure	- The NPs significantly increased both the biogas (113% enhancement) production and its quality (231% enhancement in methane production at 20 mg/L NPs); - The remaining digestate could be used as biofertilizer or soil modifier.	[67]
**Fe-Co-Cu TMNPs**	- TMNPs were synthesized by the co-precipitation method - TMNPs contained 15.46% Fe, 12.54% Co, and 25.96% Cu - NPs’ size: 34–52 nm - NPs’ shape: flowers and nanorods - Used concentrations: 10, 20, 30, 40, 50 mg/L	Palm oil mill effluent	- Cumulative biogas: 390 mL (10 mg/L), 500 mL (20 mg/L), 570 mL (30 mg/L), 400 mL (40 mg/L), 600 mL (50 mg/L), and 530 mL (0 mg/L).	[68]
**AgNPs and CuONPs**	- NPs’ size: 23.1 ± 6.9 nm (AgNPs); 25–55 nm (CuONPs) - NPs’ shape: nearly spherical - Stabilizing agent: Polyvinylpyrrolidone for AgNPs	Wastewater sludge	- Biogas generation decreased with increasing NP concentrations; - Removal of chemical oxygen demand (COD) decreased; - AgNPs had no impact on living sludge bacteria, while CuONPs decreased the percentage of living cells.	[69]
**Fe_3_O_4_ and Cu@Fe_3_O_4_ core–shell NPs**	- NPs were synthesized by the co-precipitation method - Surface area: 66.56 m^2^/g (Fe_3_O_4_ NPs) and 181.4 m^2^/g (Cu@Fe_3_O_4_) - NPs’ size: 45 nm (Fe_3_O_4_ NPs) and 143 nm (Cu@Fe_3_O_4_) - Saturated magnetization: 67.1 emu/g (Fe_3_O_4_ NPs) and 49.3 emu/g (Cu@Fe_3_O_4_) - Used concentrations: 5, 10, 20, 40, 80 mg/L	Sewage sludge	- The optimal dosage of Fe_3_O_4_ NPs and Cu@Fe_3_O_4_ NPs was 40 and 20 mg/L, respectively; - A total of 20 mg/L of Cu@Fe_3_O_4_ NPs enhanced biogas production from sewage sludge by a factor of 3.0 in comparison with using 40 mg/L of Fe_3_O_4_ NPs; - The degradation efficiencies (20 mg/L of Cu@Fe_3_O_4_ NPs) for COD_total_, TP, and VFA_total_ were 70.2, 91, and 73%.	[70]
**ZnO, Al_2_O_3_, and Fe_2_O_3_ NPs**	- NPs were obtained by the impregnation method into Polymethyl acrylate - Used quantity of NPs: 0.1, 0.2, 0.3 g	Dry wastewater sludge	- Daily biogas production was higher for Al_2_O_3_NPs and Fe_2_O_3_NPs, and lower for ZnONPs, than the control; - ZnONPs inhibited the hydrolysis of soluble proteins and polysaccharides; - The relative bacterial abundance was lower for ZnONPs; - *Methanobacterium* sp., *Methanobrevibacter* sp., and *Methanothrix* sp. increased in the presence of Al_2_O_3_NPs and Fe_2_O_3_NPs.	[71]
**ZnO NPs**	- NPs size: 80–100 nm - Concentration: 0, 5, 30 and 100 mg/g TS	Cattle manure	- Promoted the accumulation of soluble COD, soluble protein, and polysaccharides but inhibited their following fermentation and methane production processes; - Decreased the abundances of functional bacteria from 72.11% to 11.24%.	[72]
**Co_3_O_4_ NPs**	- NPs were obtained by the co-precipitation method - Spinel Co_3_O_4_ cubic structure, 100% purity - Sphere-like shape - BET surface area: 30.312 m^2^/g - Concentration: 5 mg/L	Brown algae *Cystoceira myrica* (0.5, 1, and 1.5 mg/L)	- Increased biogas production by 18%, 6%, and 59% for 0.5, 1, and 1.5 mg/L of *C myrica*; - The addition of NPs decreased the biomethane yield.	[73]
	- NPs were obtained by the co-precipitation method - NPs’ size: 15–20 nm - Irregular spherical form - BET surface area: 46.5 m^2^/g - Concentration: 1,3, 5 mg/L	Cow dung (CD control) and noxious aquatic weeds (AWs)	- The cumulative biogas production was 44.2, 55.2, 60.2, 70.2, and 69.4 L/kg Vs for CD, CD + AW, CD + AW + 1 mg/L NPs, CD + AW + 3 mg/L NPs, and CD + AW + 5 mg/L NPs, respectively; - The cumulative methane production increased by 18.05% (1 mg/L NPs), 43.4 (3 mg/L NPs), and 31.1% (5 mg/L NPs) in comparison with CD + AW; - NPs decreased the feedstock retention time during the AD process; - COD removal: up to 88.8% (3 mg/L NPs).	[74]
**TiO_2_ nanoplates**	- Square TiO_2_ nanoplates were obtained through chemical synthesis - NPs’ surface area: 9.5 m^2^/g	Cassava wastewater and sewage sludge	- Biogas production increased up to 51% on the 4th day of AD (up to 39.57 mL/g Vs) - NPs were efficient in the removal of total solid (up to 48%) and volatile solids (up to 65%), COD (up to 39%), after 21 days.	[75]
**TiO_2_ NPs**	- NPs size: 5–7 nm - Spherical shape - Concentration: 10 mg/L	Sludge wastewater	- Soluble COD removal: up to 98.1%; - Biogas production increased with 8.8%; - Methane percentage: 85.3%.	[76]
**CuO NPs**	- NPs size: 40 nm - Concentration: 100 mg/kg dry waste	Cattle manure	- CuO boosts biogas and methane production, and its size is key for faster, higher yields and to initiate methane production; - Maximum cumulative biogas: 264.9 mL/g Vs (60.9% higher than control); - Cumulative methane yield: 68.25 mL/g Vs (83.1% higher than control); - Maximum consumption rate of total volatile fatty acids: 85% compared with 65% for control; - Enhanced the abundance of hydrolytic and fermentative bacteria in phylum *Firmicutes*.	[77]
**Stainless steel nano-alloys**	- Obtained by laser ablation at 19, 26, 60 mJ/pulse - Spherical nano-alloys with size between 10 and 60 nm	Fresh cow dung	- Cumulative biogas production after 26 days: 4840 mL (26 mJ/pulse), 1575 (19 mJ/pulse), and 1690 (60 mJ/pulse); control: 2337 mL	[78]
**n-C, n-Cu_2_O, n-ZnO and n-Al_2_O_3_**	- NPs size: <100 nm for all NPs	Wastewater active sludge	- TS removal efficiency: 78.00% (n-C), 71.29% (n-Al_2_O_3_), 57.24% (n-ZnO), 30.00% (n-Cu_2_O), and 65.48% (control); - VS removal efficiency: 72.63% (control), 89.00% (n-C), 80.73% (n-Al_2_O_3_), 63.5% (n-ZnO), and 30% (n-Cu_2_O); - TCOD removal: 1.09% (control), 80.76% (n-C), 75.55% (n-Al_2_O_3_), 59.87% (n-ZnO), and 33.12% (nano-Cu_2_O); - The biogas yield increased by 20.62% (n-C) and 8.27% (n-Al_2_O_3_), while n-ZnO and n-Cu_2_O supplementation caused reductions of 34.3% and 57.3%; - The methane content increased by 11.49% (n-C) and 8.63% (n-Al_2_O_3_^)^ and decreased by 23.19% (n-Cu_2_O) and 14.8% (n-ZnO).	[79]
**SnO_2_ NPs—doped mica**	- Obtained by the co-precipitation method - Concentration: 0.03 and 0.06 mg/L	Cattle manure	- The addition of 0.03 mg/L NPs increased biogas yield by 18.1% and methane yield by 33%; - The hydrolysis stage was shorter in the AD system, and the addition of 0.03 mg/L NPs contributes to TS, VS, and COD removal.	[66]

In their study, Yadav et al. [50] focused on biogas generation through the anaerobic decomposition of food waste by incorporating graphene nanoparticles (GNPs) and multiwalled carbon nanotubes (MWCNTs). The study found that adding 100 mg/L of these carbonaceous nanomaterials significantly increased biogas production by 81.16% (GNPs) and 33.55% (MWCNTs) while improving organic matter degradation. However, increasing their concentration up to 500 mg/L led to reduced biogas production due to potential cytotoxic effects on the microbial communities. Microbial diversity analysis revealed *Firmicutes*, *Proteobacteria*, *Actinobacteriota*, and *Bacteroidota* as the dominant phyla, with carbon nanomaterials facilitating direct interspecies electron transfer.

The use of MWCNTs decorated with Fe_3_O_4_ nanoparticles (Fe_3_O_4_@CNT, 500 mg/L) in the presence of a magnetic field (12 mT, MF) has shown a 5.07% improvement in methane production compared with the control group, suggesting that the combination of magnetic materials and a magnetic field can enhance methane production in the anaerobic digestion of cellulose [80]. This treatment also increased the abundance of acetic acid-trophic methanogens, which are important for methane production. Microbial community analysis revealed variations in microbial communities with the addition of Fe_3_O_4_@CNTs; the group with Fe_3_O_4_@CNT coupled with MF exhibited the highest microbial diversity.

The addition of nano-Fe_2_O_3_ and nano-C60 (nano-fullerenes) in the process of anaerobic digestion of livestock and poultry waste liquid showed enhanced gas production, with the highest biogas output observed in the first 10 days of the experiment [81]. Specifically, nano-C60 showed a 115.8% increase in cumulative gas production, while nano-Fe_2_O_3_ achieved an 81.09% increase compared with the control group. The presence of nanoparticles, due to their high chemical reactivity, accelerated the hydrolysis process and increased the concentration of volatile fatty acids, although the pH dropped, temporarily inhibiting methane production. The microbial analysis showed that *Firmicutes* and *Bacteroidetes* dominated at the beginning of the AD process, with *Actinobacteria* and *Proteobacteria* becoming more abundant as the experiment progressed. The study concluded that nanoparticles can enhance biogas production and stabilize the anaerobic digestion process, contributing to carbon emission reduction and sustainable waste management [81].

The role of trace elements and nutrients in enhancing biogas production cannot be overlooked. Research has indicated that adding essential trace elements, such as iron and nickel, can stimulate the activity of methanogenic microorganisms, thereby increasing methane production [55,82]. The incorporation of nanomaterials that serve as carriers for these trace elements can further enhance their bioavailability and effectiveness in anaerobic digestion systems. This approach not only improves biogas yields but also supports the overall sustainability of the digestion process by guaranteeing that microbial populations have access to essential nutrients for peak performance.

One of the main obstacles in the anaerobic digestion of various substrates is the breakdown of lignocellulosic structures. The application of pre-treatment methods is an effective approach to breaking down large, complex molecules into smaller, more easily hydrolyzed structures, increasing the surface area accessible to enzymes and microorganisms. Combining these pre-treatments with metallic nanoparticles can create a synergistic effect, further enhancing the efficiency of the digestion process. Metallic nanoparticles can act as catalysts, accelerating the degradation of lignocellulose and improving microbial accessibility to organic matter [43,44,45].

The combination of 6% NaOH solution pre-treatment with magnetite nanoparticles significantly enhanced biogas and methane production from the anaerobic co-digestion of municipal solid waste and sewage sludge. The maximum biogas and methane production was achieved with the 6% NaOH solution pre-treatment combined with 110 ppm of Fe_3_O_4_ NPs, resulting in an 86% and 162% increase compared with the control, respectively. This synergistic effect also led to a substantial reduction in total solids and volatile solids, with the highest removal rates observed in the same treatment [44].

Jafari et al. [56] assessed both the environmental and economic aspects of producing biomethane through the anaerobic digestion of sewage sludge and wheat straw, enhanced by polypyrrole Fe_3_O_4_ nanoparticles and alkaline pre-treatment using sodium hydroxide (NaOH). The results demonstrated that retention time (RT) and NaOH concentration significantly influenced biomethane yields, whereas the effect of nanoparticles alone was less pronounced. Overall, the combined addition of NaOH and NPs boosted biomethane production by approximately 25–40% compared with the control. It was also noted that alkaline pre-treatment was more effective at lower NP concentrations, with its benefits diminishing as NP levels increased. Economically, the most cost-effective biogas and biomethane production were achieved with N60Na5 (60 mg/L NPs and 5% NaOH) and N20Na5 (20 mg/L NPs and 5% NaOH), reducing costs by 57% and 47%, respectively. A sustainable production scenario was identified by the authors at NP and NaOH concentrations of 50.24 mg and 24.04 mg, respectively, resulting in a 198% increase in biomethane yield and significant reductions—ranging from 86% to 95%—across human health, ecosystem quality, climate change, resource use, and economic cost compared with the control.

The application of microwave pre-treatment to meat industry sludge in the presence of Fe_2_O_3_ nanoparticles resulted in an increase of up to 35% in soluble COD, leading to a higher biogas yield [43]. This effect was attributed to the behavior of Fe_2_O_3_ nanoparticles under the electromagnetic field, where they effectively converted a significant portion of the absorbed electromagnetic energy into heat. This localized heating facilitates the breakdown of organic molecules in the sludge, enhancing solubilization and improving anaerobic digestion efficiency.

Combining thermal and alkaline pre-treatments significantly improved lignin removal, achieving up to twice the efficiency compared with a single pre-treatment method. Calcium hydroxide (Ca(OH)_2_) proved more effective in delignification when paired with thermal treatment than potassium hydroxide (KOH). The inclusion of silver nanoparticles further enhanced biogas production, with Ca(OH)_2_-treated biomass yielding 5% more biogas than KOH-treated biomass. Specifically, the methane yield for Ca(OH)_2_-treated pig manure sludge was 10% higher than for sugarcane bagasse. The highest biogas yield was observed from Ca(OH)_2_-treated PMS subjected to 30 min of autoclaving, 20% alkali concentration, and silver NPs at 5 mg/kg of solid substrate. Moreover, silver NPs did not negatively affect the anaerobic digestion process despite their known antimicrobial properties [45].

Also, the use of nano-Fe_2_O_3_ and nano-C_60_ in the anaerobic digestion process of sulfamethoxazole (SMX)-containing chicken manure led to an antibiotic degradation rate of up to 99.9% [51]. This process significantly promoted the growth of anaerobic microbiota, which in turn enhanced biogas production by up to 35% with nano-Fe_2_O_3_ and an impressive 130% with nano-C_60_. The nanoparticles not only facilitated a more efficient breakdown of the antibiotic but also contributed to optimizing microbial community dynamics, creating a more favorable environment for methane-producing bacteria. Moreover, the presence of SMX and nanoparticles had a notable impact on enzymatic activity. In particular, dehydrogenase and hydrolytic enzymes exhibited increased activity, accelerating the decomposition of organic matter and improving overall digestion efficiency [51]. In the presence of oxytetracycline, Fe_2_O_3_ nanoparticles (90 nm) incorporated in chicken manure increased the biogas production by 77% in the first 10 days of the AD process, also promoting high degradation rates of the antibiotic, up to 96.9% after 5 days of the AD experiment [83]. The size of Fe_2_O_3_ nanoparticles plays a crucial role in enhancing biogas production during the anaerobic digestion (AD) of swine manure. NPs with a size of 184 nm yielded 238.15 mL/gVS, whereas 164 nm NPs produced a value of 151.385 mL/gVS and 176 nm NPs generated 197.68 mL/gVS. The total biomethane production with 184 nm Fe_2_O_3_ NPs was 2312.98 mL for swine manure and 10,952.96 mL for co-digestion with corn straw. This enhancement is attributed to the optimal microbial diversity associated with 184 nm nanoparticles, particularly favoring key methanogenic species such as *Methanocorpusculum*, *Methanosarcina*, and *Methanomassiliicoccus* [54]. Biosynthesized Fe_3_O_4_ magnetic nanoparticles that enhanced the AD of wheat straw underwent pre-treatment with potassium hydroxide and cattle rumen fluid, leading to increased levels of volatile fatty acids, biogas production, and methane yield. Raising the NP concentration from 25 to 100 ppm resulted in a 4.2–9.9 fold, 0.33–1.5 fold, and 0.4–2.4 fold increase in total VFAs, biogas, and methane, respectively, compared with the control. This improvement is attributed to the NPs’ ability to sustain biological activity and release metal ions that support microbial communities [48].

The study [53] on the synergistic promotion of direct interspecies electron transfer (DIET) by biochar and Fe_3_O_4_ nanoparticles in the anaerobic digestion of vegetable waste revealed that the addition of Fe_3_O_4_@biochar (BC) at a concentration of 200 mg/L was optimal for enhancing biogas production and reducing VFAs accumulation. At this concentration and an organic loading rate (OLR) of 3.715 g (VS)/L·d, the biogas production rate reached 0.658 L/g (VS). Moreover, the addition of Fe_3_O_4_@BC improved the diversity and abundance of microbial communities, contributing to a more stable fermentation process, preventing acidification, and increased methane yield. Also, the incorporation of Fe_3_O_4_@BC improved the stability of the anaerobic fermentation process, effectively preventing acidification even at high OLR levels. The stimulation of the methanogenesis process and the increase in biogas yield is significantly influenced by the size and concentration of magnetite nanoparticles, as well as by the type of inoculum. Nanoparticles with medium sizes (150–480 nm) are more effective than those smaller than 150 nm or larger than 800 nm, reducing the incubation period (lag phase) by 41% and increasing the methane production by 32% [84].

The supplementation of the anaerobic co-digestion of cow manure and sewage sludge with Fe_3_O_4_ nanoparticles has been shown to significantly boost the key stages of hydrolysis, acidogenesis, and methanogenesis, as evidenced by batch experiments where an optimal nanoparticle dosage of 160 mg/L led to substantial increases in hydrolysis (up to 98.78%) and acidification (up to 76.65%), consequently facilitating a considerable rise in methane yield (up to 98.6%) and maximum production rate, with kinetic modeling further confirming the accelerated biogas generation and hydrolysis rates associated with the incorporation of Fe_3_O_4_ nanoparticles in this co-digestion process [85].

Ngema et al. [86] explored the influence of magnetite nanoparticles on the AD of municipal wastewater using an up-flow anaerobic sludge blanket (UASB) reactor, focusing on how magnetite concentration, hydraulic retention time, and organic loading rate affected bioenergy production. Introducing magnetite at concentrations ranging from 0.25 to 1 g/L revealed that even a low dose of 0.25 g/L could notably shorten the lag phase by stimulating early microbial activity, increase methane content by 40%, and significantly improve contaminant removal (91% COD, 95% color, 98.9% turbidity). Further testing with 0.5 g/L of magnetite and a 21-day HRT using more concentrated wastewater showed a threefold rise in biogas yield under a higher OLR, though with diminished pollutant removal, indicating the need for post-treatment to ensure effluent quality [86].

The anaerobic co-digestion of waste sludge with aloe vera sludge in the presence of iron nanoparticles improved both the biogas (+ 62.5%) and methane (96.9%) production. Due to their high content in minerals, vitamins, enzymes, fatty acids, and polysaccharides [87], the aloe vera wastes improve the performance of anaerobic digestion of waste sludge. Supplementation of anaerobic digesters, operating at an optimized aloe vera waste to waste sludge ratio (2:1), with Fe^0^ nanoparticles resulted in a substantial increasing of methane yield, achieving 321 mL CH_4_ gVS^−1^, a 146.9% elevation compared with the control. This enhancement is attributed to the multifaceted role of Fe^0^ nanoparticles in pH buffering, H_2_ provision for methanogenesis, redox-mediated electron transfer enhancement during acetate oxidation, and direct electron donation via corrosion-induced H_2_ generation [59].

Another study [57] explored the potential of using of both recycled and laboratory synthesized iron nanoparticles as a sustainable catalyst to enhance biogas production through anaerobic digestion. The results showed that reactors supplemented with recycled iron nanoparticles at a concentration of 50 mg/L achieved the most significant improvements, with a 123.3% increase in total biogas production and a 108.3% rise in methane yield compared with control reactors. In contrast, reactors using laboratory-grade nanoparticles showed slightly lower improvements of 114% in biogas production and 105% in methane yield. The superior performance of recycled iron nanoparticles was attributed to their ability to facilitate direct interspecies electron transfer, improve microbial activity, and stabilize the anaerobic digestion process. The nano-zero valent iron (NZV) has also been used to increase the methane yield in biogas by influencing the structure and metabolic ways of the microbial community from AD [58,88].

Yang et al. [89] demonstrated that the formation of NZV-based nanocomposites with carboxymethyl cellulose (CMC), extracellular polymeric substances (EPSs), and sodium dodecyl sulfate (SDS) represented an effective strategy to enhance both biogas production and methane yield compared with the anaerobic digestion of kitchen wastewater using only NZV. This improvement was attributed to the generation of hydrogen, particularly in the SDS-based composite, which resulted in a biogas yield of 510 mL/gVS with a methane content of 93%. Furthermore, the application of NZV-based composites led to a significant increase in methanogenic bacterial populations, particularly *Methanothrix* and *Methanolinea*, with a relative growth of 43.6%. These microbial communities play an important role in the final stage of anaerobic digestion by facilitating the conversion of acetate and hydrogen into methane [90].

Spherical Co and Ni nanoparticles (NPs), with diameters ranging from 17 to 28 nm, boosted biogas and methane production during the anaerobic digestion of slurry. The introduction of trace metals in NP form not only shortened the lag phase but also accelerated the peak biogas and methane production, highlighting a clear biostimulator effect on methanogenic activity throughout the startup and hydraulic retention time (HRT) of the process. Specifically, the addition of 1 mg/L Co NPs (28 ± 0.7 nm) enhanced biogas and methane yields over 50 days of HRT, while a higher concentration of 2 mg/L exhibited toxicity, reducing production. In contrast, biogas and methane production increased proportionally with a Ni NP concentration of up to 2 mg/L (17 ± 0.3 nm), where Ni NPs effectively stimulated methanogenic bacteria and boosted methane content [39]. Also, the addition of CoNPs could stimulate the early stages of AD, affecting, in a positive manner, system stability and acetoclastic methanogenesis [91]

Another study [64] explored the impact of a mixture of metal nanoparticles (Fe, Ni, and Co) on the anaerobic digestion of poultry litter and the subsequent uptake of these nanoparticles by plants fertilized with the AD effluent. Conducted over 278 days, the results revealed that NP inclusion significantly enhanced methane (CH_4_) production by 23.7% while reducing hydrogen sulfide (H_2_S) emissions by 56.3%. Increased methane yield was due to the catalytic properties of the nanoparticles, which enhanced microbial activity and facilitated greater organic degradation. Additionally, the reduced H_2_S production was due to the binding of sulfur to the metal nanoparticles, which prevented the formation of toxic H_2_S gas. However, the study also highlighted that the NPs anaerobic digestion effluent contained significantly higher concentrations of NPs—ranging from 1160% to 19,400% higher than the control. When this effluent was used as a fertilizer in a hydroponic system growing buttercrunch lettuce, the plant biomass absorbed 21.0% to 1920% more NPs compared with the control. Despite the significant uptake, no visible signs of phytotoxicity or adverse effects on plant growth were observed. The elevated concentrations of essential nutrients like nitrogen and phosphorus in the NPs’ anaerobic digestion effluent also contributed to improved lettuce biomass. In contrast, the incorporation of Sn-Mn-Fe trimetallic nanoparticles, as demonstrated by Akar et al. [67], in the anaerobic digestion of fresh cow manure, yielded even more substantial improvements, achieving a 113% increase in biogas production and an impressive enhancement of up to 231.2% in methane generation, although after 45 days retention time. Furthermore, given that the constituent metals of these trimetallic nanoparticles are essential micronutrients for biological systems, the resulting digestate holds promising potential for applications as a biofertilizer or soil amendment.

The addition of CoNPs [91] at a concentration of 7 mg/g (VS) increased the stability of sewage sludge anaerobic digestion, almost doubled the biogas production compared with control (232 vs. 132 mL/g (Vs)), reduced volatile solids by 16%, and increased methane content by 74.3%, positively affecting AD stability and acetolactic methanogenesis.

The incorporation of silver nanoparticles (AgNPs) into the anaerobic digestion of sewage sludge notably enhanced methane production by 15% and increased volatile solid (VS) removal by 25%, without adversely affecting process stability. The AgNPs enhanced methane production kinetics, shortening the lag phase and increasing the maximum rate of methane generation. While methane content remained stable across treatments, hydrogen sulfide (H_2_S) levels were significantly reduced, improving biogas quality. The composition of the microbial community shifted significantly, showing a five-fold increase in *Methanosarcina*, a key methanogen, in the AgNPs reactors. Despite the antimicrobial nature of silver, neither AgNPs nor silver nitrate (AgNO_3_) hindered the anaerobic digestion process [60]. The results of this study contradict the results of Abdulsada et al. [69] regarding the impact of Ag and CuO nanoparticles on the anaerobic digestion process, which demonstrated significant inhibition of this process and a reduction in biogas production. This discrepancy suggests that the influence of metal nanoparticles on anaerobic digestion processes is complex and dependent on specific factors, such as the concentration, size, and type of nanoparticles used, as well as the experimental conditions. Potential interactions between nanoparticles and anaerobic microbiota or the composition of the organic substrate may contribute to the variability of the results.

The use of mesoporous SiNPs and Ag@SiNPs at the AD process of landfill leachate led to an increase in biogas production with 14% for SiNPs and 37% for Ag@SiNPs, simultaneously with the reduction in COD by 58% and vs. by 65%. The Ag@SINPs improved the activity of bacteria responsible for the methanogenic process [61].

The impact of cobalt oxide nanoparticles (Co_3_O_4_-NPs) on anaerobic digestion and biogas/methane production was investigated in different studies [73,74]. The results showed enhanced biogas yields upon nanoparticle supplementation, although the optimal dosage and the extent of improvement varied depending on the substrate (cow dung and water hyacinth [74] co-digestion versus brown algae [73]). Specifically, the first study [74] found a maximum biogas enhancement at 3 mg/L of Co_3_O_4_-NPs, alongside increased methane yield and microbial viability, leading to a techno-economic analysis indicating a substantial net energy content and profit. In contrast, the second study [73], optimizing process parameters for brown algae digestion using response surface methodology, identified 5 mg/L of Co_3_O_4_-NPs as optimal for biogas production, while surprisingly, maximum biomethane generation occurred without nanoparticle addition. Nevertheless, a techno-economic assessment still demonstrated financial benefits for biogas production with nanoparticle addition, albeit at a lower net energy and profit compared with the first study. The use of photoactivated Co_3_O_4_ NPs (5 mg/L) in dry-anaerobic co-digestion of cow manure with whey (mixing ratio 2:1) increased both the biogas and methane production by 35% and 16%, respectively [66].

The impact of NiO, CoO, and Fe_3_O_4_ nanoparticles, both individually and in combination, on the anaerobic digestion of primary sludge and waste-activated sludge mixtures from a municipal wastewater treatment plant revealed a dose-dependent relationship concerning methane and biogas production, as well as total solids and volatile solids removal [63]. Notably, lower concentrations of NiO and CoO enhanced the AD process, while higher levels proved inhibitory; conversely, Fe_3_O_4_ nanoparticles did not negatively impact digestion across the tested concentrations and even promoted efficiency at a mid-level (10 mg/L), approaching the optimal performance observed with 1 mg/L NiO. Furthermore, beneficial treatments accelerated the time to peak biogas and methane generation, with 1 mg/L NiO and a specific mixture (1 mg/L NiO, 1 mg/L CoO, and 10 mg/L Fe_3_O_4_) demonstrating the highest efficiency in biogas and methane yields alongside organic matter removal. Regarding biogas quality, lower NP concentrations had minimal effect on hydrogen sulfide (H_2_S) content, whereas a significant reduction in H_2_S (13.36–52.57%) was observed at the highest concentration (100 mg/L).

## 6. Research Gaps and Future Prospects in Using NPs as Catalysts in Ad

**Figure 6:**
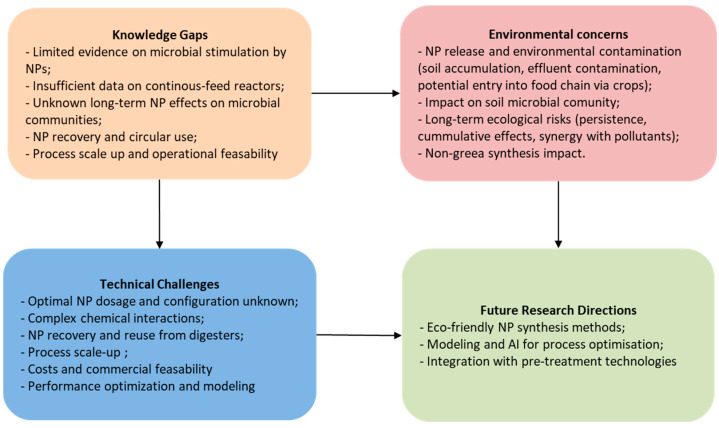
Challenges of using nanoparticles in anaerobic digestion.

Using nanoparticles in anaerobic digestion shows promise but comes with big challenges (Figure 6).

Anaerobic fermentation is a biochemical process that involves a large and diverse microbial community for the decomposition of organic matter. Although many scientific works have highlighted the potential of NPs in favoring methanogenesis, the area of synergism between anaerobic microorganisms and NPs still requires detailed investigations.

There is relatively insufficient evidence to confirm that fermentative microorganisms capable of converting organic substrates of various types into methane can be stimulated efficiently and economically by supplementing the mass with nanomaterials [92]. On the other hand, not much is known about the toxic potential of some NPs that reach the environment with the agricultural use of fermented sludge. The role of microorganisms in maintaining ecological balance is essential for environmental health as they break down complex organic compounds into simple forms that become accessible nutrients for plants and other organisms. Microorganisms also ensure the return of essential nutrients to the soil, thus contributing to maintaining its fertility. NPs can severely disrupt microbial communities, causing both structural changes through oxidative stress and functional changes, thereby negatively affecting nutrient bioavailability [93]. NPs can impact microbial metabolism through chemical interactions with natural organic compounds and toxic pollutants in the environment.

Carbon-based nanoparticles have been proved to be effective in anaerobic fermentation processes as they facilitate enzyme immobilization, shorten the lag phase duration, and increase the rate of methanogenesis, thereby increasing biogas production and methane concentration. However, the dosage of carbon-based NPs was not significant in terms of the amount of biogas obtained in comparative laboratory experiments [94]. Many studies show that anaerobic digesters supplemented with carbon-based NPs can operate stably and efficiently in batch operations. Lab-scale tests usually performed in batch experiments cannot be extrapolated to anaerobic processes performed in continuous flow; little attention has been paid to research on the effect of NPs on AD in continuous-feed pilot reactors [95]. The usual mode of operation for industrial bioreactors is the continuous or semi-continuous feeding of the organic substrate. Therefore, laboratory results are very useful for understanding phenomena and mechanisms at the micro scale, but they do not help practically in implementation because the study conditions in bioreactors of a few liters to tens of liters are not realistic compared with those existing in continuous or semi-continuous reactors with volumes of thousands of cubic meters [42]. Knowing that experimental research is mainly carried out on experimental models or batch-fed pilot plants, it is necessary to study and establish how the continuous dosing of NPs in scaled-up bioreactors will be performed, whether NPs will be fed together with the substrate or separately, with the same frequency or less frequently [96].

Another challenge that requires special attention is the collection and separation of nanomaterials from aqueous solution, since many of these nano-sized compounds cannot be properly separated from the digester but end up in the wastewater treatment plant or bio-waste disposal sites; contamination of sewage sludge with toxic NPs may be harmful for the soil [92]. Despite the important role of metal nanoparticles in enhancing biogas production through improved microbial activity and organic degradation, there are valid concerns about their potential toxicity to the environment and food safety due to NP accumulation in crops. Some studies have shown that the effluent from anaerobic digestion systems treated with NPs contained significantly higher concentrations of metal nanoparticles, leading to substantial uptake by plants grown with the effluent [64]. This increases the potential for nanoparticles to enter the food chain, posing potential risks to human health and soil ecosystems. Furthermore, the long-term effects of NP exposure on plant metabolism and soil microbial communities remain unclear. Consequently, while NPs show promising advantages for renewable energy production, vigilant monitoring and regulation are important to avoid unintended environmental contamination and safeguard the safety of agricultural products. Moreover, further research is needed to understand the mechanisms through which these nanoparticles affect the biochemical processes involved in anaerobic digestion and to identify potential optimal conditions that minimize the negative effects on biogas production. A challenge for future research could be the characterization and possible uses of the digestate, taking into account the presence and possible toxicity of nanomaterials.

To achieve the maximum energy yield in anaerobic reactors operated with biomass supplemented with nanoparticles, it is helpful to identify and use appropriate optimization and modeling techniques for operational parameters [97]. Research on the mathematical modeling of optimal theoretical conditions for particular types of substrates is an investigation pathway that can be approached and exploited. Also, experiments on biomethane potential with different types of nanoparticles added at different stages of the process can provide valuable indications on the bacterial species on which the tested NPs prove to be most effective. It is beneficial to check the impact of nanoparticles with different configurations, sizes, and concentrations on microbial activity and on the overall performance of the whole AD process (hydrolysis, acidogenesis, acetogenesis, and methanogenesis). The multitude of NP synthesis techniques allow for obtaining and testing a wide variety of compounds tailored to meet specific requirements. Since the methods for the synthesis of NPs are varied and their ratio to the biomass mass is still to be optimized, we can state that there is some lack of coherence in the confident adoption of this pre-treatment option.

Future research should also focus on investigating the role of magnetic nanomaterials, which are a very promising class of compounds, in increasing biogas production and sustainable waste treatment [98]. Inhibitory effects were observed at higher doses of magnetic nanoparticles, highlighting the need for careful dosage optimization to avoid negative effects on anaerobic digestion processes. Finding strategies to prevent magnetic nanoparticles from adhering to the walls of steel digesters and to keep them in suspension in order to achieve their intended purpose is crucial. This could be achieved by applying external magnetic fields to activate the nanoparticles, but research is also needed in this direction, primarily considering the need for personnel safety.

Very little information is available on the kinetics of AD, as well as on the unfortunate conditions that lead to the inhibition of methane production as a result of the generation of toxic chemicals in the system. The NPs’ long-term impact on bacterial metabolism and microorganisms’ dynamics within the bioreactor are some aspects that worth being carefully studied. The investigation and integration of computer-aided techniques such as modeling, simulation, and AI algorithms (Machine Learning, Fuzzy Logic, Genetic Algorithms, etc.) for the operational optimization of a biogas reactor through the selection of the most favorable reaction conditions (temperature, pressure, mixing rate, biomass-to-NP ratio, etc.) to maximize the production of desired products with the least material investment can contribute significantly to the practical implementation of NP catalysts in anaerobic digestion processes.

An important barrier to the implementation of the use of NPs as catalysts in commercial anaerobic reactors is their production cost. Hassaan et al. [99] indicate the price of NPs as the main aspect to consider for their commercial use to be profitable. Much research is still required to move from the level of scientific results to that of implementation in industrial facilities, at a cost-competitive price.

**Figure 7:**
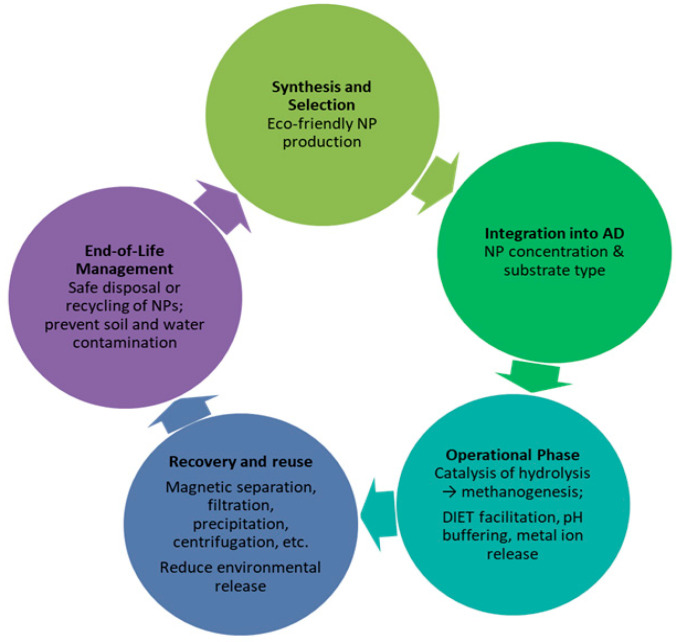
Life cycle of nanoparticles in anaerobic digestion.

From a life cycle perspective, efficiency gains must be balanced against sustainability concerns, including the environmental fate of nanoparticle components in the digestate, their potential accumulation in soil, and the need for safe recovery or recycling pathways to prevent environmental hazards (Figure 7). Sustainable deployment requires green synthesis methods, closed-loop nanoparticle use, and integration with renewable feedstocks, ensuring that intensified biogas production aligns with both short-term energy recovery and long-term environmental responsibility.

The development of eco-friendly synthesis methods for metal nanoparticles, particularly those suitable for industrial-scale applications, represents a promising direction in nanotechnology. Among these, radiochemical synthesis has emerged as a viable and sustainable approach, offering significant advantages in terms of cost reduction and precise control over nanoparticle characteristics such as size, stability, and dispersion. Studies by Lungulescu et al. [100,101] have highlighted the potential of this method, demonstrating its efficiency and scalability. By minimizing the use of hazardous chemicals and energy-intensive processes, eco-friendly not only aligns with environmental sustainability goals but also enhances the feasibility of large-scale nanoparticle production for various industrial and technological applications.

## 7. Conclusions

The integration of nanotechnology into anaerobic digestion processes holds significant promise for enhancing biogas production efficiency, process stability, and overall sustainability. As detailed in this study, nanoparticles, particularly those based on metals and metal oxides, can act as effective catalysts, facilitate critical biochemical reactions, and accelerate microbial metabolism during all stages of anaerobic digestion. Their unique physico-chemical properties, such as high surface-area-to-volume ratio and enhanced reactivity, allow for better interaction with both substrates and microbial communities, ultimately leading to increased methane yields and reduced retention times. Research indicates that the careful incorporation of NPs based on iron, nickel, cobalt, silver, and their oxide counterparts, TiO_2_, SnO, carbonaceous nanomaterials, etc., can improve the degradation of complex organic compounds, stimulate methanogenesis, and mitigate the inhibitory effects of ammonia and volatile fatty acids.

Additionally, synergistic effects have been observed when combining NPs with pre-treatment methods such as alkaline hydrolysis, microwave irradiation, or enzymatic conditioning, further enhancing substrate biodegradability and process efficiency. Despite these advancements, the transition from laboratory to industrial-scale application requires a cautious approach due to potential environmental risks and uncertainties related to nanoparticle toxicity, long-term stability, and their fate in the digestate.

Moreover, comprehensive assessments on cost-effectiveness, optimal dosing, and potential bioaccumulation impacts in agricultural systems are essential. Therefore, while nanoparticle-assisted anaerobic digestion represents a transformative frontier for biogas technologies, further interdisciplinary research is needed to address existing gaps, optimize nanoparticle use, and develop scalable, safe, and economically viable solutions for sustainable bioenergy generation.

## Figures and Tables

**Table 1:** Pre-treatment methods to enhance biomass-to-biogas conversion.

Category	Method Type	Specific Techniques
Physical Pre-treatment	Mechanical	Milling; grinding; chipping; extrusion
	Thermal	Hydrothermal; steam explosion; liquid hot water (LHW)
	Irradiation	Microwave irradiation; ultrasonication; γ-irradiation
	Electrical	High voltage pulse discharge
Chemical Pre-treatment		Acid treatment; alkaline treatment; mixed treatment (acid for hemicellulose; alkali for lignin removal); ionic liquids; deep eutectic solvents; alkaline hydrogen peroxide; ammonia fiber explosion (AFEX); supercritical CO_2_ explosion
Biological Pre-treatment	Microbial	Using bacterial strains isolated from soil, fish gut, rumen fluid, etc.
	Enzymatic	Enzymes from fungi and bacteria: Laccases, Glucanases, Cellobiose, Xylanases, Pectinase, and Manganese
Combined Pre-treatment	Physico-chemical (e.g., thermal conditioning and chemical treatment; alkaline and microwave pre-treatment; ultrasound and peroxidation; mechano-chemical pre-treatment); bio-physical pre-treatment (e.g., yeast extract added to the substrate and thermal pre-treatment); combined acid–alkaline pre-treatment	

## Data Availability

Not applicable.
